# Alginate Oligosaccharides Inhibit Fungal Cell Growth and Potentiate the Activity of Antifungals against *Candida* and *Aspergillus* spp

**DOI:** 10.1371/journal.pone.0112518

**Published:** 2014-11-19

**Authors:** Anne Tøndervik, Håvard Sletta, Geir Klinkenberg, Charlotte Emanuel, Lydia C. Powell, Manon F. Pritchard, Saira Khan, Kieron M. Craine, Edvar Onsøyen, Phil D. Rye, Chris Wright, David W. Thomas, Katja E. Hill

**Affiliations:** 1 Department of Bioprocess Technology, SINTEF Materials and Chemistry, N-7465 Trondheim, Norway; 2 Advanced Therapies Group, Tissue Engineering and Reparative Dentistry, Cardiff University School of Dentistry, Cardiff, CF14 4XY, United Kingdom; 3 AlgiPharma AS, Industriveien 33, N-1337 Sandvika, Norway; 4 Centre for NanoHealth, Systems and Process Engineering Centre, College of Engineering, Swansea University, Swansea, SA2 8PP, United Kingdom; Institute of Microbiology, Switzerland

## Abstract

The oligosaccharide OligoG, an alginate derived from seaweed, has been shown to have anti-bacterial and anti-biofilm properties and potentiates the activity of selected antibiotics against multi-drug resistant bacteria. The ability of OligoG to perturb fungal growth and potentiate conventional antifungal agents was evaluated using a range of pathogenic fungal strains. *Candida* (n = 11) and *Aspergillus* (n = 3) spp. were tested using germ tube assays, LIVE/DEAD staining, scanning electron microscopy (SEM), atomic force microscopy (AFM) and high-throughput minimum inhibition concentration assays (MICs). In general, the strains tested showed a significant dose-dependent reduction in cell growth at ≥6% OligoG as measured by optical density (OD_600_; *P*<0.05). OligoG (>0.5%) also showed a significant inhibitory effect on hyphal growth in germ tube assays, although strain-dependent variations in efficacy were observed (*P*<0.05). SEM and AFM both showed that OligoG (≥2%) markedly disrupted fungal biofilm formation, both alone, and in combination with fluconazole. Cell surface roughness was also significantly increased by the combination treatment (*P*<0.001). High-throughput robotic MIC screening demonstrated the potentiating effects of OligoG (2, 6, 10%) with nystatin, amphotericin B, fluconazole, miconazole, voriconazole or terbinafine with the test strains. Potentiating effects were observed for the *Aspergillus* strains with all six antifungal agents, with an up to 16-fold (nystatin) reduction in MIC. Similarly, all the *Candida* spp. showed potentiation with nystatin (up to 16-fold) and fluconazole (up to 8-fold). These findings demonstrate the antifungal properties of OligoG and suggest a potential role in the management of fungal infections and possible reduction of antifungal toxicity.

## Introduction

The increase in invasive fungal infections over the last decade is a growing cause for concern as they are associated with high mortality rates [1]. The likely reasons for this increase include greater use of immunosuppressive therapy, selective pressure through broad-spectrum antibiotic use, medical and prosthetic device-related infections [2], as well as the spread of HIV/AIDS and an increasingly ageing population [1], [3]. *Candida albicans* and *Aspergillus fumigatus* are the most common species associated with fungal invasive diseases, but the importance of other *Candida* species and filamentous fungi like *Scedosporium* and *Fusarium* [4] is becoming apparent. In addition to *C. albicans*, *C. glabrata*, *C. parapsilosis*, *C. tropicalis* and *C. krusei*, together account for 92% of the detected cases of candidemia [5]. In aspergillosis, after *A. fumigatus*, *A. flavus* is the most common causative species [6].

Amphotericin B has for many years been the first line of treatment for fungal infections despite its nephrotoxic side effects. Amphotericin B and polyene resistance is occasionally reported, but until now, has had limited clinical importance [4]. However, azole antifungals such as fluconazole, itraconazole and voriconazole, although effective antifungal agents, have shown development of resistance [7]; it being well-recognised among *Aspergillus* species in particular. Echinocandins, a newer class of antifungals which are generally well tolerated, are considered to be a potential first line treatment for many patients due to their activity against azole-resistant strains [8], [9]. In general, the clinical value of many common antifungal therapeutics is limited by toxicity, and considerable efforts are being made to reduce this toxicity in some of the conventional antifungal drugs. For example, less toxic lipid formulations of amphotericin B have now been produced and approved for clinical use [10].

Another strategy for enhancing efficacy and reducing drug toxicity is the use of combined therapies. Several *in* *vitro* studies performed using combinations of antifungals have shown synergistic effects, demonstrating the potential of this strategy [11], [12], [13], [14]. An alternative approach has been to potentiate the effect of antifungals through the use of different non-antibiotic compounds, such as peptides [15], [16], ion-chelators [17], essential oils [18], [19] or secondary metabolites of plant and microbial origin [20], [21], [22]. Combining antifungals with antibiotics such as colistin (an antibiotic in current use with significant toxicity issues) has also recently proved successful [23]. To date, however, none of these products have progressed as far as clinical use.

The novel alginate oligomer OligoG is an oligosaccharide enriched from sodium alginate polysaccharides and composed predominantly of α-l-guluronic acid (>96%), possessing only a small percentage of the β-d-mannuronic acid isomer (<4%). OligoG has recently been shown to perturb multi-drug resistant bacteria by influencing biofilm formation and reducing resistance to antibiotic treatment [24]. In the present study, we tested this alginate oligomer in combination with conventional antifungal treatment (selected polyenes, azoles and allylamines) against a panel of *Aspergillus* and *Candida* strains, to determine if this potentiation was evident against fungal pathogens.

## Materials and Methods

### Fungal strains, alginate oligomers and antibiotics

The strains used in this study represent both culture collection strains and clinical isolates and are shown in Table 1. The alginate oligomer, OligoG, used in the study was prepared, purified and characterized as described previously [24] and the antifungals employed were pharmaceutical grade (Sigma-Aldrich).

**Table 1:** Strains used for susceptibility testing and their source.

Strain designation	Source
**Yeasts**	
*Candida albicans* ATCC 90028	Blood
*Candida albicans* CCUG 39343^1^	Human faeces
*Candida parapsilosis* ATCC 22019T^2^	Coeliac patient
*Candida krusei* 141/03	Pseudomembraneous candidosis
*Candida krusei* 249/03(2)	Ulceration
*Candida lusitaniae* 994/01(2)	Candidosis suspension
*Candida tropicalis* 12	Vaginal
*Candida tropicalis* 519468	Urinary
*Candida tropicalis* 250/03	Pseudomembraneous candidosis
*Candida tropicalis* T2.2	Oral
*Candida glabrata* ATCC 2001	Faeces
**Molds**	
*Aspergillus niger* CCUG 18919 (ATCC 16404)^2^	Blueberry
*Aspergillus fumigatus* CCUG 17460	Unknown
*Aspergillus flavus* CCUG 28296	Shoe sole

1 Resistant to 5-flucytosine, fluconazole, itraconazole.

2 Recommended by CLSI as reference strains for antifungal susceptibility testing.

### Preparation of freezer-stock cultures and growth characterization of the test strains

*Candida* strains were grown at 34°C for 48 h on Yeast Mold-agar (YM-agar, Difco). One to three fungal colonies from the plates were grown in 6 mL YM-broth at 34°C for 14 h before freezing in 6% glycerol at −80°C. *Aspergillus* strains were grown at 30°C for 96 h on YM-agar. Spores and aerial mycelia were then cut from the agar, suspended in 1 mL YM-broth and dispersed with glass beads (1 mm) in a mini bead-beater (2 min). Glycerol was added to a final concentration of 10% and the suspension was frozen at −80°C. Each batch of frozen stock culture was then characterized in separate growth experiments (results not shown) using Mueller-Hinton broth (MH, Lab114, LabM) and RPMI with 0.2% glucose (RPMI-1640, Sigma-Aldrich) to determine the minimum amount of inoculum giving satisfactory growth after 48 h under conditions relevant for the determination of minimum inhibitory concentration assays (MICs) as described below. This inoculum procedure was used to reduce inter-experimental variation in the bioassays. For characterization of growth in the two different media, strains were grown with static incubation in 384-well microplates (30 µL per well, Nunc 242757) and optical density (OD_600_) measured between 24–96 h using a Beckman Coulter Paradigm microplate reader. Prior to OD measurements, the microplates were shaken at 1800 rpm (2.5 mm amplitude) for 2 mins.

### Susceptibility testing by robotic minimum inhibitory concentration (MIC) assay

The ability of alginate oligomers to potentiate the activity of selected antifungals against the *Candida* and *Aspergillus* test strains was studied by susceptibility testing using high-throughput robotic screening (HTS) as described previously [24]. Alginate oligomers were dissolved in medium to 1.25 times the desired assay concentrations (2, 6 and 10%). Selected antifungals from different classes included: nystatin, amphotericin B (polyenes), fluconazole, miconazole and voriconazole (azoles) and the topical antifungal, terbinafine (allylamine). The *Candida* strains were tested against nystatin, fluconazole and terbinafine, (one from each class) whilst the *Aspergillus* strains were tested against all six antifungals. Two-fold serial dilutions of antifungals were made in medium with different concentrations of OligoG, and the solutions were placed in four parallel wells in 384-well micro plates (30 µL per well). Serial dilutions were performed with a Tecan Genesis RSP 200 liquid handling workstation equipped with an 8-channel pipetting tool, using sterile disposable 200 µL barrier tips. Into each well in the 384-well assay plates 7.5 µL, of the medium inoculated with frozen stock culture of the relevant strains (described above) was added. The microtitre plates were placed in plastic bags and incubated in a Thermo Cytomat 2 450 S robotic incubator without shaking at 34°C. Optical density (OD_600_) was measured at specific time points between 24–96 h. The microplates were shaken at 1800 rpm (2.5 mm amplitude) for 2 mins prior to taking the absorbance readings. The readings made at 48 h were used for all strains except for the fluconazole test with *C. tropicalis* 519468 and T2.2. For these strains OD_600_ readings after incubation for 36 h were used, since the readings after 48 h were inconclusive.

Synergy for the antifungal drug/OligoG MIC combinations tested was determined from the Fractional Inhibitory Concentration Index (FICI; [25]) where FICI≤0.5 is indicative of synergy. The FICI was calculated from the MIC of the drug in combination, divided by the MIC of the drug acting alone.

### Germ Tube Assay

Overnight cultures of *C. albicans* CCUG 39343, ATCC 90028, *C. tropicalis* 519468 and *C. glabrata* ATCC 2001 were prepared in Sabouraud-dextrose broth (SAB, Oxoid) and incubated at 37°C. *C. glabrata* as a non-hyphae producer was the negative control. One mL of culture was then washed twice with phosphate buffered saline (PBS) and the resulting pellet resuspended in 500 µL PBS, to obtain approximately 5×10^6^ cells/mL. Serial dilutions (100 µL) were spiral plated onto Sabouraud-dextrose agar (SAA; Oxoid) to calculate colony forming units per mL (CFU/mL). Donor horse serum (500 µL; TCS Biosciences Ltd) supplemented with 0, 0.2, 0.5, 2, 6 or 10% OligoG was inoculated with 50 µL of the washed candidal suspension, and incubated for 2 h at 37°C. Following incubation, 100 µL of the candidal/serum suspension was serially diluted and spiral plated onto SAA to calculate post incubation CFU/mL. The remaining suspension was washed with 0.9% NaCl (x3) to remove OligoG, and resuspended in 200 µL PBS. The percentage number of cells with hyphal growth was calculated using a Neubauer haemocytometer under phase contrast microscopy. Light microscopy images of the different hyphal growth were also taken.

### LIVE-DEAD Staining of *Candida* biofilms

An overnight culture of *C. tropicalis* 519468 was grown in RPMI (Invitrogen). Biofilms were grown in 8-well chamber slides (BD Falcon), using 35 µL of the overnight culture per well (10^7^ cells), followed by the addition of either 350 µL of RPMI or 350 µL of 2% OligoG solubilized in RPMI. These were grown for 24 h at 37°C, with gentle rocking. The supernatant was then removed and the biofilm stained with the LIVE/DEAD BacLight Bacterial Viability Kit (Invitrogen, Paisley, UK) containing SYTO 9 dye and propidium iodide, prior to being imaged under a fluorescent microscope (Olympus Provis AX70).

### Scanning Electron Microscopy Imaging

*C. tropicalis* 519468 was grown overnight in RPMI, and the culture was diluted in the same medium to attain 0.4×10^7^/mL cells. Biofilms were then formed on thermanox slides (Agar Scientific) in the bottom of 12-well Cellstar plates (Greiner Bio-One, Stonehouse, UK) using 1 mL of culture incubated with gentle rocking at 37°C for 4 h. Each well was then washed (x3) with pre-warmed RPMI. Then, 1 mL of either 2, 1, 0.5 µg/mL fluconazole and/or 2% OligoG solubilized in RPMI was added to each well and further incubated with gentle rocking at 37°C for 24 h. The supernatant was then removed and each well immersed in 2.5% glutaraldehyde for 1.5 h and then washed thoroughly with distilled water. Biofilms were then frozen (−20°C) after addition of 1 mL distilled water to each well. Once frozen, the well plates were then freeze-dried for 24 h. The thermanox slides were then removed from the well plates and imaged using an Hitachi S4800 scanning electron microscope.

### Atomic Force Microscopy Imaging

*Candida* cultures for atomic force microscopy (AFM) imaging were prepared following a similar protocol to that of Murillo et al. [26]. *C. tropicalis* 519468 was grown as a shaken overnight culture in SAB. The overnight culture (10 mL) was centrifuged at 2,100×*g* for 10 mins and resuspended in fresh SAB at 37°C to attain 10^7^/mL cells (OD_520_). Biofilms were formed in polystyrene petri dishes (60 mm×15 mm) using 6 mL of 10^7^ cells in SAB, and incubated with gentle rocking at 37°C for 30 mins. The biofilms were then thoroughly washed (x3) in pre-warmed media and 6 mL of fresh media and/or 2% OligoG and fluconazole (1 mg/L) added followed by incubation with gentle rocking at 37°C for a further 90 mins. The use of this shorter biofilm growing time was necessary to optimize AFM imaging. The biofilms were rinsed twice with de-ionised water and dried at room temperature for 1 h before imaging with a Dimension 3100 AFM instrument (Bruker) using a scan speed of 0.4 Hz. Mean surface roughness (Ra) measurements were used to investigate the impact of the cell treatments on the morphology of the fungal cell wall by measuring a 1 µm^2^ area at the centre of each fungal cell observed within 50 µm^2^ tapping mode images.

### Statistical Analysis

Statistical analysis was performed using GraphPad Prism3 (GraphPad software Inc, California, USA). A paired T-test with 95% confidence intervals was used to test the significance in the differences in growth at various OligoG-concentrations and in the mean number of cells producing hyphae following incubation with OligoG. Where the rules of a paired T-test were not obeyed, a Mann-Whitney test was performed. The non-parametric Kruskal-Wallis one-way analysis of variance was used for analysis of the surface roughness data. *P*<0.05 was considered significant.

## Results

### Growth characteristics of *Candida* and *Aspergillus* spp

Initial studies were performed to characterize the growth of *Candida* and *Aspergillus* strains in 384-well plates in RPMI and MH. According to standardized protocols these media are recommended for antibiotic susceptibility testing in yeast and bacteria respectively [27], [28]. For all strains tested, the cell mass obtained after incubation for 24–48 h (which is the recommended duration of incubation before MIC determination) in RPMI was lower than that observed in MH, as shown for *C. tropicalis* 519468 and *A. flavus* (Figure S1). The primary objective of the present study was to explore the effects of OligoG on fungal growth, either alone or in combination with antifungal agents. Even though MH is used mostly for bacterial susceptibility testing, we chose to use this broth for our studies since all our test strains exhibited better growth characteristics under the relevant cultivation conditions in MH. However, parallel experiments were performed in RPMI with selected strains to ensure that the effects observed were not exclusive to MH (Figure S2). *Aspergillus* strains reached a higher cell density (1.3–1.7 OD_600_) than the *Candida* strains (0.5–0.8 OD_600_) in MH.

**Figure 1:**
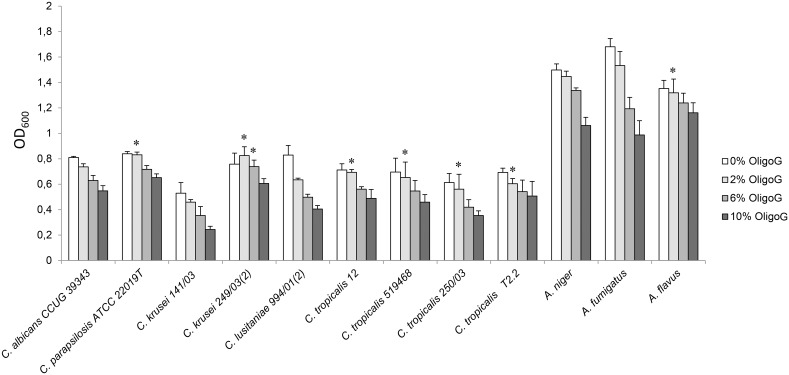
Effect of increasing concentrations of OligoG (0, 2, 6, and 10%) on cell densities. Cultivation in Mueller-Hinton broth for 48 h at 34°C for various *Candida* and *Aspergillus* species. Error bars represent standard deviation from the mean (n≥4). (*, data not significantly different from control results; *P>*0.05).

Addition of OligoG to MH reduced the growth of all the fungal strains tested in a concentration dependent manner (Figure 1). Hence, the effect was most pronounced with 10% OligoG leading to a 15–50% reduction in cell density after 48 h incubation. Growth of selected strains of *Candida* (n = 5) and *Aspergillus* (n = 3) in RPMI also showed a reduction in OD_600_ of 8–35% with 10% OligoG (Figure S2), showing that this effect was not media-dependent. Analysis of the growth kinetics showed that the specific growth rate (µ) in MH was only slightly reduced by the addition of OligoG. Typically for *A. flavus* and *C. tropicalis* 519468 growth rates of µ = 0.22/t (without OligoG) and µ = 0.19–0.2/t (with 10% OligoG) were observed.

### Germ Tube Assay

**Figure 2:**
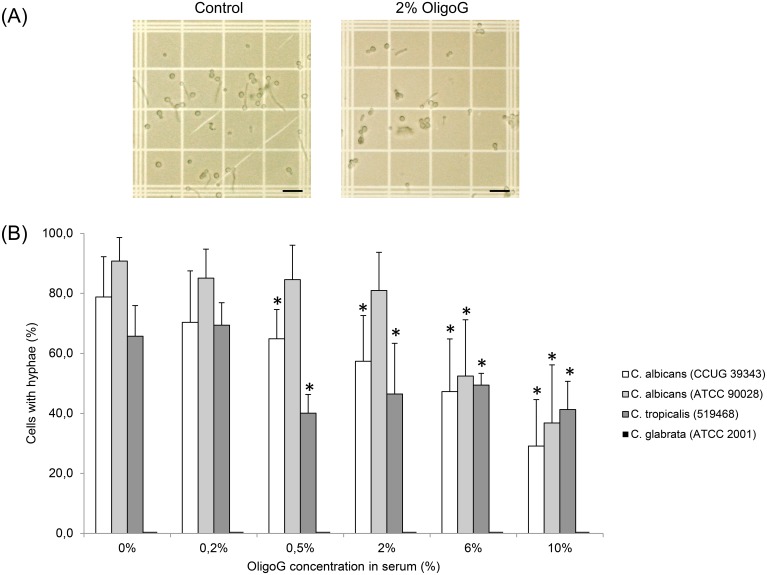
Germ tube assays. (A) Light microscopy images of *Candida albicans* (CCUG 39343) cells grown with/without the presence of OligoG, (Scale bar is 100 µm). (B) Percentage of *Candida* cells producing hyphae for four different strains grown for 2 hours in the presence of OligoG (0, 0.2, 0.5, 2, 6 and 10%). *Candida glabrata* as a non-hyphae producer was the negative control. *indicates significantly different from the control, (*P*<0.05).

Light microscopy images of the germ tube assay revealed that OligoG had a pronounced effect on the hyphal growth of *C. albicans* CCUG 39343 (Figure 2A) demonstrating a reduced number of cells producing hyphae following treatment with OligoG. The actual concentration of OligoG required to produce an effect however, was species-dependent. The percentage number of cells with hyphal growth decreased significantly using ≥0.5% OligoG for *C. albicans* 39343 and *C. tropicalis* 519468, and ≥6% OligoG for *C. albicans* 90028 when compared to the control (Figure 2B; *P*<0.05). The pH of the *Candida* cultures in serum remained between pH 7.2 and 7.5 throughout, showing that the observed effects were not pH related.

### Effect of OligoG on *C. tropicalis* biofilms using fluorescence microscopy, scanning electron microscopy (SEM) and atomic force microscopy (AFM)

**Figure 3:**
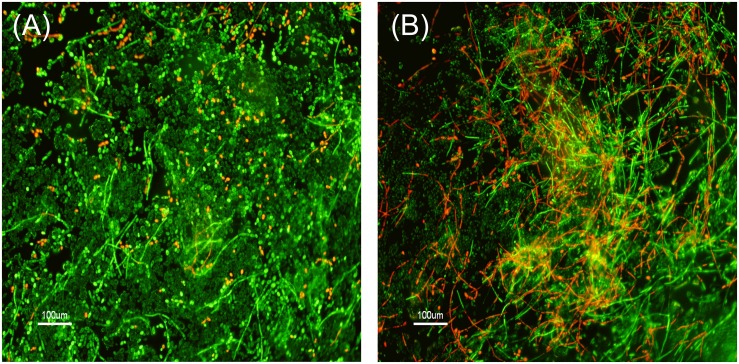
LIVE/DEAD fluorescence imaging of *Candida tropicalis* 519468. Biofilms were grown for 24 h at 37°C showing vital live cells (green) versus dead cells (red). (A) Untreated control. (B) 2% OligoG.

**Figure 4:**
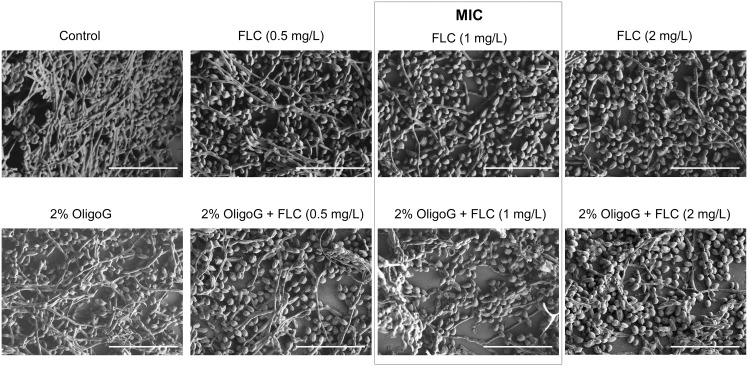
Scanning electron microscopy of *Candida tropicalis* 519468 (x 1.20 K) treated with 2% OligoG with/without fluconazole (FLC). FLC was used at concentrations of 0.5, 1 and 2 µg/mL (equivalent to ‘below’, ‘at’ and ‘above’ the MIC value respectively). Scale bar is 40 µm.

**Figure 5:**
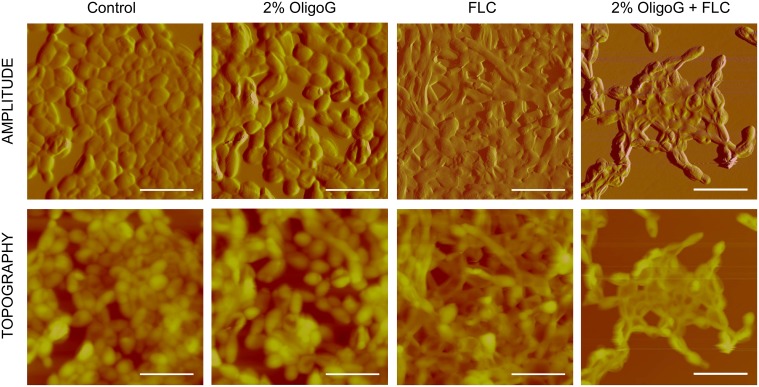
AFM imaging of *Candida tropicalis* 519468 grown on polystyrene with/without 2% OligoG and/or fluconazole (FLC). FLC was used at 1 mg/L (equivalent to the MIC) with more rounded cells (post OligoG treatment), more flattened cells (post fluconazole treatment) and both flattened, and “wrinkled” cells (post combination treatment) apparent. Z scale of 7.5 µm. Scale bar is 15 µm.

LIVE/DEAD staining (Figure 3), SEM (Figure 4) and AFM images (Figure 5) all revealed that *C. tropicalis* 519468 biofilms grown in the presence of 2% OligoG showed distinct differences in biofilm structure compared to the control. OligoG treatment caused increased cell and hyphal death (LIVE/DEAD staining), which in turn resulted in a more ‘open’ and less densely formed biofilm matrix (SEM). The SEM images also appeared to confirm the germ tube assay findings that *C. tropicalis* grown in the presence of OligoG produced less hyphae. Fluconazole used at concentrations equivalent to ‘below’, ‘at’ and ‘above’ the MIC value in conjunction with 2% OligoG revealed a potentiation effect on the inhibition of *C. tropicalis* (Figure 4). The combined treatment (at every fluconazole dose) produced a more porous/open structure of the biofilm (with less biomass and more water channels) when compared to fluconazole treatment alone.

AFM imaging (Figure 5), showed less marked alterations in the structure of the OligoG-treated biofilms than that observed with LIVE/DEAD and SEM imaging and reflected the use of less mature biofilms (2 h), which were necessary to optimise the AFM imaging (compared to 24 h in the other imaging experiments). Nevertheless, there were clear morphological changes in response to treatment, with either more rounded cells (after OligoG treatment), or more flattened cells (after fluconazole treatment). However, the combination treatment resulted in reduced cell and hyphal growth, while also showing the most distinctive morphological changes, with cells appearing both flattened, and “wrinkled” when compared to fluconazole or OligoG treatment alone (Figure 5). There was no significant difference in surface roughness (Ra) measurements between the control and 2% OligoG treated samples (*P*>0.05). However, there was a significant increase in Ra for both the combined fluconazole and 2% OligoG, and fluconazole alone treatments (*P*<0.001) compared to the control (Figure S3).

### The effect of OligoG on potentiation of antifungals against *Candida* and *Aspergillus* spp

OligoG was able to potentiate the effect of selected antifungal agents commonly used to treat fungal infections (Table 2 and Table 3). For the three *Aspergillus* strains, a reduction in MIC was found for all antifungal agents tested. This decrease was similar for all antifungals (≤4-fold) except nystatin, for which the greatest decrease was observed (up to 16-fold). All the *Candida* strains showed potentiation with nystatin (up to 16-fold with *C. parapsilosis*) and fluconazole (up to 8-fold with four strains). In addition, the majority of the *Candida* test strains appeared resistant to terbinafine (MIC>32 mg/L), with the exception of *C. lusitaniae*, *C. parapsilosis* and *C. krusei*. Of all the *Candida* spp., only *C. lusitaniae* showed potentiation with terbinafine (4-fold).

**Table 2:** MIC of antifungals alone and with increasing concentrations of OligoG (2, 6, 10%) for a range of *Aspergillus* spp.

MIC (mg/L) at indicated OligoG concn (%)																				
	Nystatin				Amphotericin B				Miconazole				Voriconazole				Terbinafine			
Isolate	0	2	6	10	0	2	6	10	0	2	6	10	0	2	6	10	0	2	6	10
*A. niger* CCUG 18919	8	**4**	**2**	**0.5**	0.188	0.188	**0.094**	**0.047**	1	**0.5**	**0.25**	**0.25**	0.25	**0.063**	**<0.03**	**<0.03**	0.5	**0.25**	**0.063**	**0.125**
*A. fumigatus* CCUG 17460	8	8	**4**	**2**	0.75	**0.375**	**0.375**	**0.188**	4	**2**	**2**	**2**	0.125	0.125	**0.063**	**<0.03**	4	4	4	**2**
*A. flavus* CCUG 28296	8	8	**4**	**2**	0.75	**0.375**	**0.375**	**0.188**	1	**0.25**	**0.25**	**0.25**	0.063	0.063	**<0.03**	**<0.03**	0.125	**0.063**	**<0.03**	**<0.03**

Bold numbers indicate a Fractional Inhibitory Concentration Index (FICI)≤0.5; indicative of synergy [25].

**Table 3:** MIC of antifungals alone and with increasing concentrations of OligoG (2, 6, 10%) for a range of *Candida* spp.

	MIC (mg/L) at indicated OligoG concn (%)											
	Nystatin				Fluconazole*				Terbinafine			
Isolate	0	2	6	10	0	2	6	10	0	2	6	10
*C. albicans* CCUG 39343	8	8	**4**	**2**	16	16	**8**	**4**	>32	>32	>32	>32
*C. parapsilosis* ATCC 22019T	8	**4**	**1**	**0.5**	2	2	**0.5**	**0.25**	2	2	2	2
*C. krusei* 141/03	8	**4**	**2**	**2**	128	**64**	**64**	**64**	>32	>32	>32	>32
*C. krusei* 249/03(2)	8	8	8	**4**	8	**4**	**2**	**1**	4	4	8	4
*C. lusitaniae* 994/01(2)	8	8	**2**	**1**	0.5	**0.25**	**<0.13**	**<0.13**	8	**4**	**4**	**2**
*C. tropicalis* 12	8	8	**4**	**4**	1	1	**<0.13**	**<0.13**	>32	16	>32	32
*C. tropicalis* 519468	16	**8**	**4**	**2**	1	1	**0.25**	**<0.13**	>32	>32	>32	>32
*C. tropicalis* 250/03	8	**4**	**2**	**1**	8	**4**	**2**	**2**	>32	>32	>32	>32
*C. tropicalis* T2.2	8	8	**4**	**2**	0.5	**0.25**	**<0.13**	**<0.13**	>32	>32	>32	32

*MIC values of fluconazole for *C. tropicalis* strains (519468 and T2.2) were determined at 36 h.

Bold numbers indicate a Fractional Inhibitory Concentration Index (FICI)≤0.5; indicative of synergy [25].

## Discussion

The aim of this study was to perform a screen to determine both the direct effect of OligoG on a range of *Candida* and *Aspergillus* species and, indirectly, its ability to potentiate the activity of conventional antifungal therapies. The potentiation effects of OligoG were investigated with antifungal agents from different chemical classes; with polyenes, azoles and allylamines being employed in these assays. The antifungals which were studied included those agents most commonly prescribed for human and veterinary use. From a mechanistic and novel therapy perspective, it was of interest to determine whether any potentiation effects of antifungals seen with OligoG were similar for the entire selected range of *Candida* and *Aspergillus spp*., whilst recognising that all antifungals tested were not currently deemed clinically relevant for the test strains that were utilized.

This *in* *vitro* study clearly demonstrates that the novel alginate oligomer, OligoG, was able to modulate both fungal growth and fungal biofilm formation. Moreover, OligoG was also shown to potentiate the activity of a range of antifungal agents, giving up to a 16-fold reduction in MIC values. Given the toxicity of many antifungals, these findings represent a potential clinical benefit in the management of fungal infections. This is supported by similar observations for OligoG in potentiating the activity of antibiotics against multi-drug resistant gram-negative bacteria [24].

Resistance to antifungal agents is commonplace. The majority of the *Candida* strains tested in this study were resistant to terbinafine. A large variation in susceptibility towards terbinafine among different clinical *Candida* isolates has previously been described [29] and resistance has been linked to overexpression of the efflux pump genes *CDR1* and *CDR2* from the ABC transporter gene family [30]. The polyenes such as amphotericin B bind to ergosterol, the major sterol in the fungal cell membrane, and form pores, thereby causing membrane damage. In contrast, azoles target sterol biosynthesis by inhibiting the sterol 14 α-demethylase, the product of the *ERG11* gene. Resistance to fluconazole has been associated with point mutations and increased *ERG11* expression or over-expression of efflux-pump genes *CDR1*, *CDR2* and *MDR1* [31]. Importantly, resistance of *Candida* spp. to classic triazole antifungal agents is increasing. The MIC testing of the *Candida* strains demonstrated potentiation (up to 8-fold) of antifungal azoles.

Several efflux pumps of *A. fumigatus* have also been identified. However, their involvement in drug resistance is less well established and little is known about how their expression is regulated [31]. Recently, a deletion mutant for the transcription factor SrbA in *A. fumigatus* was found to confer susceptibility to fluconazole, which is not normally active against this pathogen [32]. Unsurprisingly therefore, there was little effect of fluconazole on the *Aspergillus* strains tested; two being resistant (MIC>128) while the *A. flavus* strain showed only a low level of potentiation (2-fold) with OligoG.

Although there is some controversy surrounding the pathogenicity of *Aspergillus* spp. and the decision to treat infections caused by this genera in some indications (i.e. lung infection in cystic fibrosis patients) these arguments are strongly influenced by the immune status of the host, clinical signs of infection and patient microbiology [33]. Overuse of antifungal agents, antifungal resistance and drug costs are all problems for these patients. The ability of OligoG to reduce the MIC and thereby potentiate the effect of antifungal agents *in* *vivo*, may provide wider treatment options in the management of resistant fungal lung infections, which are an increasing problem with triazoles.

In addition to the potentiation activity of OligoG, it clearly showed antifungal activity when used alone. The modification of biofilm structure, together with growth inhibition, and hyphae effects were evident in all of the imaging techniques (conventional light and fluorescence microscopy, SEM and AFM) employed in this study. These methods demonstrated that the altered growth characteristics were associated with distinct morphological changes, which subsequently resulted in biofilm disruption, increased cell death, and reduced hyphal formation. In previous studies, inhibition of bacterial growth by OligoG was associated with marked decreases in cell motility as seen in bacterial swarming assays [24], [34]. These earlier observations support the current findings that OligoG has a direct effect on the invasive, hyphal growth phase of *Candida* spp.

AFM is increasingly being used to study the effect of antimicrobial agents on the cell surface [24], [34]. Recently using AFM, we showed strong binding of OligoG to the cell surface of the pathogenic bacteria *Pseudomonas aeruginosa*, which remained bound even after hydrodynamic shear [35]. Although similar antimicrobial effects were observed with fungal pathogens, there was no apparent similarity in the binding of OligoG to the fungal cell wall. Nevertheless, morphological changes were clearly evident. While these studies are not directly comparable (organisms were in different growth states, planktonic versus biofilm), it would suggest that different mechanisms of action are involved in the antimicrobial effects observed in bacterial and fungal pathogens. This is unsurprising given the contrasting differences in cell wall structure and charge. The fungal cell wall is principally composed of three main components: β-glucans (microfibrillar polymers of glucose; ∼60%), chitin (∼2%) and mannoproteins (∼39%) [36]. As a means of combating potential cell lysis, the composition of the cell wall can be altered using a ‘compensatory mechanism’ which is activated in response to changes in their immediate environment such as cell wall perturbing agents or mutations. This allows the cell wall to be remodeled [37] and can result in an increased level of chitin [38]. The response to such cues can effectively direct changes in fungal growth, cell wall mass, ultrastructure, elasticity and adhesion, enzyme production and pathogenicity [39]. While it is not known whether OligoG induces this kind of remodeling effect, it is a charged molecule and so could influence, or be influenced by, molecular changes in the cell wall.

The observation that OligoG inhibits hyphae formation is particularly relevant in light of recent co-infection studies with *Staphylococcus aureus* and *C. albicans* [40]. Both organisms are commonly isolated bloodstream pathogens, often found as co-infecting agents. They are also both capable of forming biofilms, and show increasing evidence of antimicrobial resistance, thereby representing a significant and growing problem in the management of polymicrobial infections. Specific binding of *S. aureus* to the hyphae of co-cultured *C. albicans* (via Als3p binding) has been shown to enable tissue infiltration and subsequent deep tissue infection by *S. aureus* [40]. Undoubtedly, antimicrobials capable of targeting both bacteria and yeasts, such as OligoG, have a distinct advantage for such polymicrobial infections.

Although a precise molecular mechanism of action has yet to be established, it is thought that the synergistic and antimicrobial activity of OligoG in disrupting the biofilm matrix/architecture could potentially permit: a) better access for antimicrobial agents and/or host innate defenses to biofilm-embedded organisms and, b) previously dormant (drug-tolerant) cells to move into a more drug-susceptible phase of growth. The current *in* *vitro* observations would appear to support some of these hypotheses, but will need to be tested with appropriate *in* *vivo* models.

Clearly further studies are needed to determine the precise molecular mechanisms of OligoG in microbial infections. Notwithstanding, the antifungal effects and potentiating activity observed in this study represent considerable promise for the clinical utility of OligoG in the treatment and management of fungal infection and reduction of antifungal toxicity in clinical practice. Phase I and IIa studies have demonstrated that OligoG is safe for human use, *in* *vivo* testing having shown no intolerance to inhaled doses of up to 540 mg/day (https://ClinicalTrials.gov, NCT00970346 and NCT01465529; EudraCT, 2009-009330-33). OligoG is currently being developed for topical wound application, and studies are ongoing for the use of OligoG as an inhaled therapy for cystic fibrosis.

## Supporting Information

Figure S1 Growth of (A) *C. tropicalis* 519468 and (B) *A. flavus* at 34°C in Mueller-Hinton broth (open squares) and RPMI (solid squares).
(TIF)

Figure S2 Effect of increasing concentrations of OligoG (0%, 2%, 6%, and 10%) on cell densities after cultivation in RPMI broth for 48 h at 34°C for various *Candida* and *Aspergillus* species. Error bars represent standard deviation from the mean (n≥4). (*, data not significantly different from control results; *P>*0.05).
(TIF)

Figure S3 Mean surface roughness expressed as (Ra) ± standard error. *indicates significantly different from the control. FLC, fluconazole.
(TIF)
