# Supplementary figures and images for: Alginate Oligosaccharides Inhibit Fungal Cell Growth and Potentiate the Activity of Antifungals against Candida and Aspergillus spp
Source: PLoS One. 2014 Nov 19;9(11):e112518. doi: 10.1371/journal.pone.0112518 (PMC4237368; doi:10.1371/journal.pone.0112518)

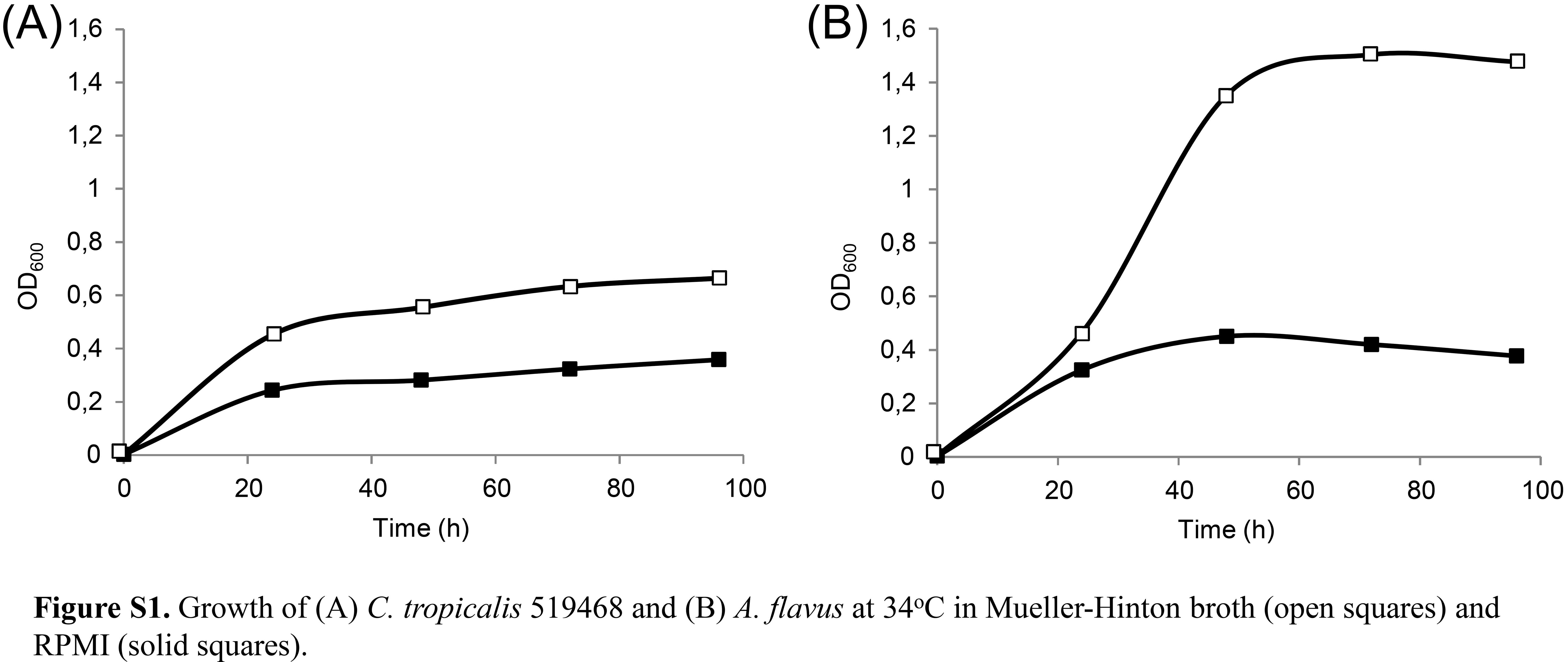

Supplement: Figure S1 — Growth of (A) C. tropicalis 519468 and (B) A. flavus at 34°C in Mueller-Hinton broth (open squares) and RPMI (solid squares). (TIF) [file pone.0112518.s001.tif]

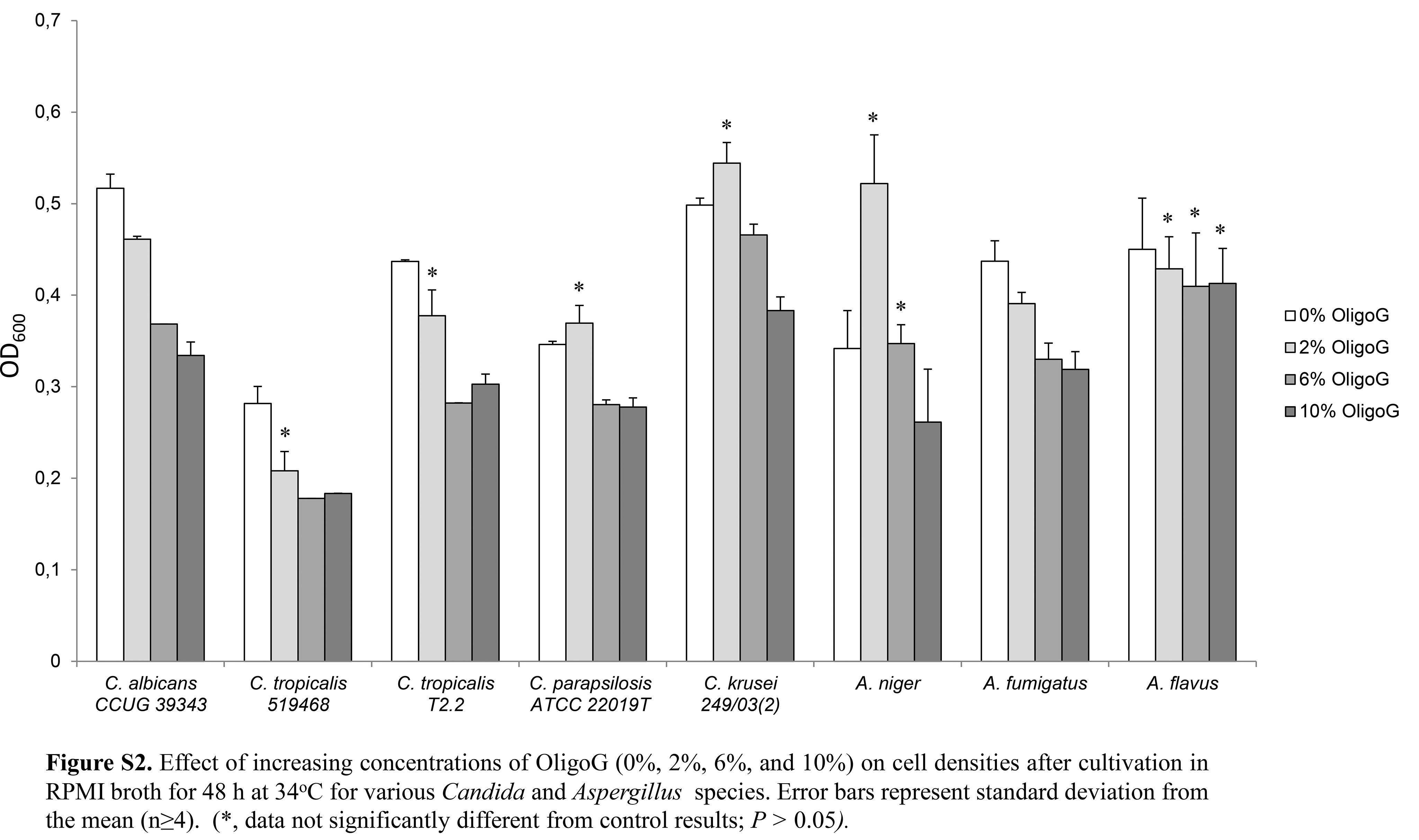

Supplement: Figure S2 — Effect of increasing concentrations of OligoG (0%, 2%, 6%, and 10%) on cell densities after cultivation in RPMI broth for 48 h at 34°C for various Candida and Aspergillus species. Error bars represent standard deviation from the mean (n≥4). (*, data not significantly different from control results; P>0.05). (TIF) [file pone.0112518.s002.tif]

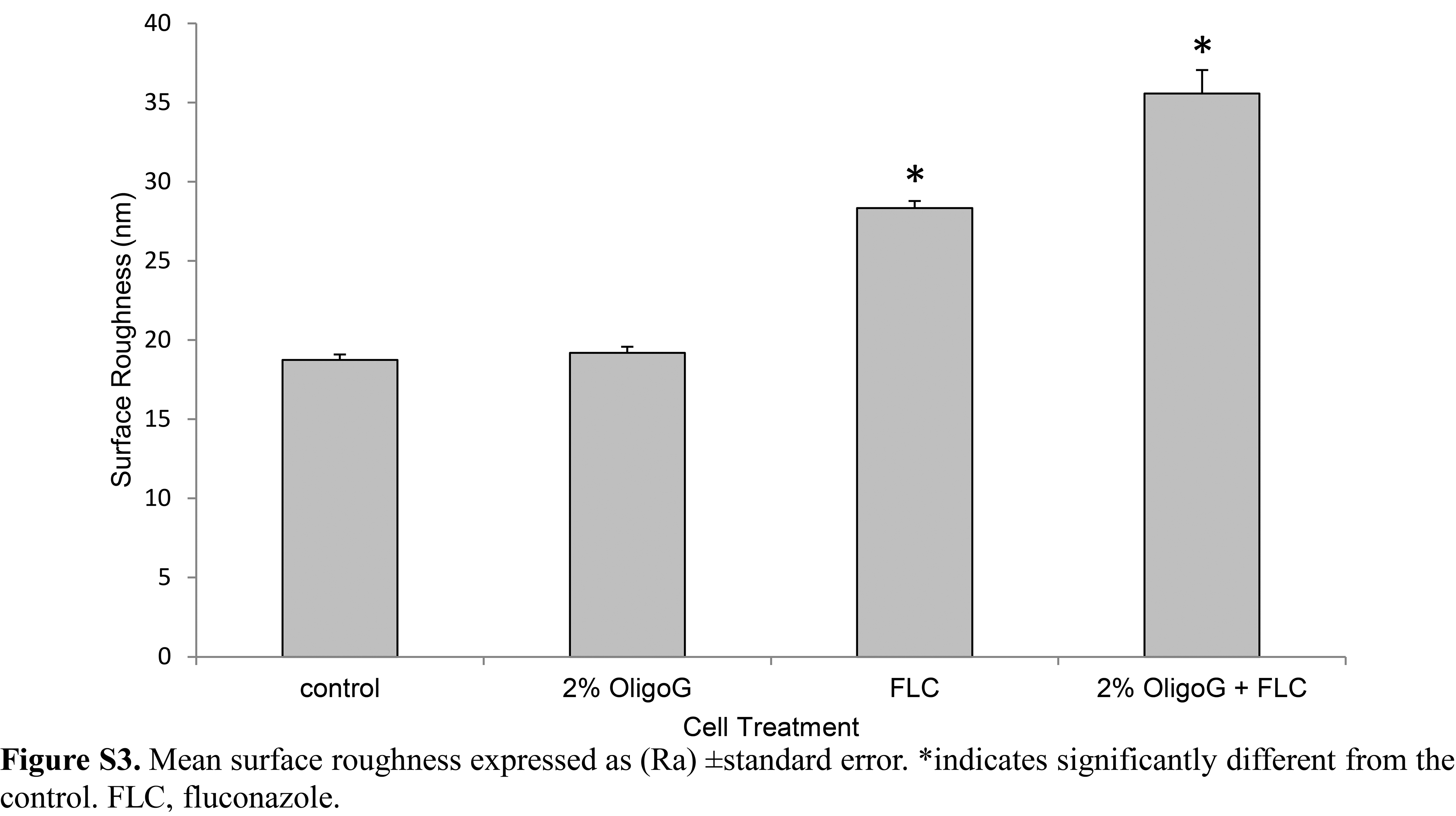

Supplement: Figure S3 — Mean surface roughness expressed as (Ra) ± standard error. *indicates significantly different from the control. FLC, fluconazole. (TIF) [file pone.0112518.s003.tif]
